# Super-rapid race for saving lives by developing COVID-19 vaccines

**DOI:** 10.1515/jib-2021-0002

**Published:** 2021-03-25

**Authors:** Anusha Uttarilli, Sridhar Amalakanti, Phaneeswara-Rao Kommoju, Srihari Sharma, Pankaj Goyal, Gowrang Kasaba Manjunath, Vineet Upadhayay, Alisha Parveen, Ravi Tandon, Kumar Suranjit Prasad, Tikam Chand Dakal, Izhar Ben Shlomo, Malik Yousef, Muniasamy Neerathilingam, Abhishek Kumar

**Affiliations:** Institute of Bioinformatics, International Technology Park, Bangalore 560066, India; Manipal Academy of Higher Education (MAHE), Manipal 576104, Karnataka, India; Department of Biotechnology, School of Life Sciences, Central University of Rajasthan, Bandarsindri, Kishangarh 305817, Rajasthan, India; Institute for Experimental Surgery, University of Rostock, Rostock D18057, Germany; School of Biotechnology, Jawaharlal Nehru University, New Delhi 110067, India; Centre of Environmental Science, Institute of Interdisciplinary Studies, University of Allahabad (A Central University), Allahabad, Uttar Pradesh, India; Genome & Computational Biology Lab, Department of Biotechnology, Mohanlal Sukhadia University, Udaipur 313001, Rajasthan, India; Program of Emergency Medicine, Zefat Academic College, Safed 13206, Israel; Department of Information Systems, Zefat Academic College, Zefat 13206, Israel; Galilee Digital Health Research Center (GDH), Zefat Academic College, Zefat 13206, Israel

**Keywords:** COVID-19, SARS-CoV-2, vaccine development

## Abstract

The pandemic of coronavirus disease 2019 (COVID-19) caused by the severe acute respiratory syndrome coronavirus 2 (SARS-CoV-2) has affected millions of people and claimed thousands of lives. Starting in China, it is arguably the most precipitous global health calamity of modern times. The entire world has rocked back to fight against the disease and the COVID-19 vaccine is the prime weapon. Even though the conventional vaccine development pipeline usually takes more than a decade, the escalating daily death rates due to COVID-19 infections have resulted in the development of fast-track strategies to bring in the vaccine under a year’s time. Governments, companies, and universities have networked to pool resources and have come up with a number of vaccine candidates. Also, international consortia have emerged to address the distribution of successful candidates. Herein, we summarize these unprecedented developments in vaccine science and discuss the types of COVID-19 vaccines, their developmental strategies, and their roles as well as their limitations.

## 1 Introduction

Our World is now facing a once-in-a-century pandemic, i.e., coronavirus disease 2019 (COVID-19), caused by SARS-CoV-2 [1]. After the first reports of the disease in December 2019 from Wuhan, China, the World Health Organization (WHO) has reported more than 75 million active COVID-19 cases with 1.68 million deaths by December 20, 2020 [1]. Lack of a cure to the disease forced many countries to opt for a complete lockdown of movement of goods and people, which caused a severe plunge in the economy [2]. However, there was a rapid surge in the global infection of COVID-19 with the death toll reaching above one million by mid-October 2020. The soaring number of COVID-19 cases could be attributed to the non-compliance of social distancing guidelines, improper use of face masks, and lifting of lockdown in several countries. Besides, the temperature drop during winter appears to induce a second peak of COVID-19 infection in several countries across Europe [1], [2]. This poses a serious threat to human life and massive loss to the global economy.

Even though several antiviral agents like hydroxychloroquine, remdesvir, favipiravir etc., are used to treat COVID-19, they do not have a strong scientific evidence of efficacy in humans. Also, these drugs have severe adverse effects. With the persistent increase in the number of cases with multiple peaks, COVID-19 vaccines are eagerly awaited. Vaccines when administered into the human body enable the immune system to recognize the antigens of the microorganisms and trigger a strong immune response by producing the antibodies against the pathogens. This blocks the replication of the pathogen on infection and thus prevents development of the disease. Thus, vaccine development is highly imperative for preventing the outspread of SARS-CoV-2 infection and helps in reducing the morbidity and mortality of the COVID-19.

This novel coronavirus was first isolated by Chinese research groups based on their findings in the Wuhan COVID-19 patients. The genomic sequence of SARS-CoV-2, characterized by several researchers, has been made available in public databases [3], [4], [5]. In the beginning of May 2020, more than 120 vaccines were in the pipeline throughout the world, and at least six groups had started injecting the formulation into volunteers [3]. According to WHO (report dated 02-March-2021), there are 76 candidate vaccines in preclinical and 182 in clinical trials [6]. Most of these vaccines use complete viral proteins or the spike protein to induce immunity. Herein, we discuss the process and progress of different types of vaccines against COVID-19.

## 2 Stages of vaccine development

**Figure 1:**
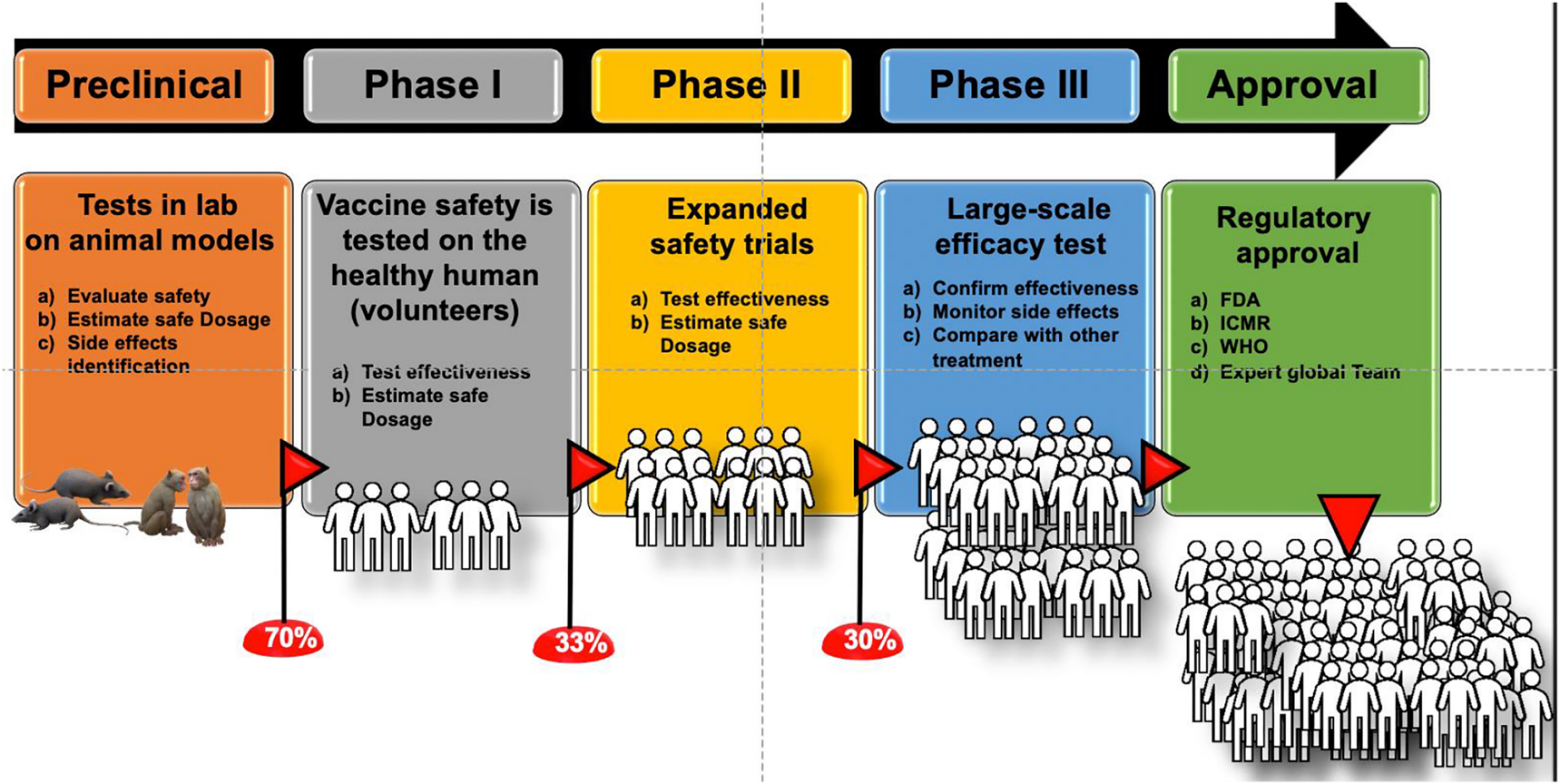
Stages of vaccine development. Vaccines requires largely five major steps, starting with preclinical tests in animal models, phase I involves limited number of human individuals and each of the next steps needs exponible increments of human individuals and it decreases change of success to reach to next levels and only limited numbers of vaccines are approved for public use by regulatory agencies of different countries.

A successful COVID-19 vaccine should be extremely safe and pure along with high efficacy and potency rates. In this context, a vaccine candidate should undergo three phases of clinical trials in humans. The vaccine developmental pipeline progresses sequentially: phase I, phase II and phase III, followed by its licensure. Phase IV studies help in continuous monitoring of the safety and immunogenicity of the candidate vaccines. We have summarized vaccine developmental landscape in Figure 1. The advantages and limitations of each of these vaccines are listed in Table 1.

**Table 1:** Advantages and disadvantages of each vaccine strategy.

Type of vaccine	Advantages	Disadvantages
Live attenuated	(i) Most immunogenic(ii) Confer long-term immunity after only one or two doses [7](iii) Can induce herd immunity [7](iv) Development of trained immunity [8]	(i) Antibody-dependent enhancement (ADE) of infection and disease [9](ii) Risk of unregulated pathogen replication [7](iii) May not be suitable for immunocompromised people and pregnant women [7]
Whole inactivated	(i) High stability(ii) Can be given in immunocompromised people [7]	(i) Weaker immune response than live-attenuated vaccines [10](ii) Require multiple doses and adjuvants [10]
Bacterial vector	Safer [10]	Require multiple doses and adjuvants [10]
Viral vector	Safe and potent at low doses [11]	Transgene-specific response may be dampened by immune responses to antigenic targets within the vector itself [11]
Protein subunits	(i) Low incidence of adverse reactions	May need adjuvants [7]
	(ii) Non-infectious [7]	
Nucleic acid based	(i) Mimics infection(ii) Easy manipulation of antigen(iii) Economical production [12]	(i) Administered foreign DNA may persist for a long period(ii) Administered foreign DNA may interact with the host [12]
Synthetic peptide	(i) Low incidence of reactions	May need adjuvants [13]
	(ii) Economical production	
	(iii) Non infectious	

### 2.1 Exploratory stage

Scientists work on natural or synthetic antigens that might help prevent or treat a disease. It may take 2–4 years to come up with a candidate vaccine. The vaccine research involves the identification and isolation of the protective antigens of a specific pathogen, methods for DNA cloning, the creation of new vector systems, and the development and immunologic evaluation of new adjuvant systems.

### 2.2 Pre-clinical stage

The candidate vaccine is tested on tissue-culture or cell-culture systems and animals. The safety and immunogenicity are assessed; modifications to the antigen made and different methods are tested for 1–2 years. Animal models provide information about the protective effects, antigen recognition, safety and toxicology aspects of the vaccine.

Drug developers in the US submit an investigational new drug (IND) application to FDA before beginning clinical research. Each country has its own regulating body. In India, the Central Drugs Standard Control Organization (CDSCO) is the national regulatory authority (NRA) for pharmaceuticals or drugs and medical devices. CDSCO acts under. The drug and cosmetics act, 1994 and rules 1995 and is responsible for the drug approval, clinical trials, proposing the standards for drugs and quality control of the imported drugs [14].

#### 2.2.1 Phase I trials

From the doses obtained on the preclinical animal studies, researchers find out how much of a maximal dose of a drug/vaccine the human body can tolerate. These phase I trials usually aim to find the best vaccine dosage with minimal or no side effects [6].

Phase Ia studies are small trials in healthy, immunocompetent naïve adults who are at low risk of acquiring a vaccine-relevant infection (determined by serology, exposure, and travel history). Phase Ib studies may be conducted in different age or population groups closer to the target population to assess possible differences in dose, safety, vaccine schedule, or route of administration [15].

Phase I trials can be either open-label or blinded and additionally either non-randomized or randomized. More than one adjuvant with the same vaccine antigen is studied. The candidate vaccine is administered to 20–80 normal human adults to assess its safety and to determine the type and extent of immune response that it provokes. Data is gathered about how a drug interacts with the human body. Information on the side effects associated with increased dosage is obtained. This information helps to determine the best route and dosage to administer the drug to limit risks and maximize possible benefits. These generally take over several months of activity and 70% of the drugs move to the next phase [16].

#### 2.2.2 Phase II trials

Phase II studies are randomized controlled trials performed on hundreds of subjects to study the candidate vaccine’s immunogenicity, safety, schedule of immunizations, proposed doses, and method of delivery. These phase II studies assess the impact of multiple variables on immune response, such as age, ethnicity, gender, and presence of maternal or pre-existing antibodies (in infants). However, for a vaccine being developed for infants a step-down approach is usually followed wherein trials are conducted sequentially in adults, adolescents, children, and infants [6]. Identification of an immune correlate of protection is identified, facilitates the interpretation of results in future clinical studies with immune response as end points [6].

Phase II trials can also provide preliminary information on protective efficacy through human challenge studies, wherein healthy participants are deliberately infected with the pathogen. Such studies are commonly referred to as phase IIa studies and are appropriate only for selected diseases wherever it is scientifically and ethically justified, where the pathogen does not cause lethal infection and is not resistant to available treatment, and a complete and successful cure can be obtained.

These steps are important to refine research questions, develop research methods, and design new phase III research protocols. The duration of these studies may last up to two years. Approximately 33% of the candidate vaccines go through to phase III stage.

#### 2.2.3 Phase III trials

These are randomized placebo controlled double blind trials performed with thousands of subjects to study the efficacy and tolerability of the vaccine [6]. The results show long-term or rare side effects because these studies are larger and longer in duration. These take from one year to four years for completion. These studies are essential for registration and approval till marketing of a vaccine and further, to assess the effect of the final formulation. A successful phase III trial could lead to approval for human administration. Phase I to III trials are placebo-controlled, double-blinded, and randomized study by design [6].

#### 2.2.4 Phase IV trials

Phase IV trials are performed after the vaccine is in the market. These are optional studies that drug manufacturers may conduct to test the vaccine for safety, efficacy, and other potential uses [6].

### 2.3 Accelerated vaccine development strategies

It may take up to 10 years for a vaccine until it reaches the public in the conventional path. But the unprecedented situation in recent times, has led to the deployment of accelerated vaccine development strategies. Even as scientists sequenced the genome of the novel coronavirus and developed several promising vaccine candidates with extraordinary speed [17]. Moderna’s mRNA-based SARS-CoV-2 candidate entered phase I trials on March 16, less than 10 weeks after the first genetic sequences were released [18].

Bioinformatics tools have placed crucial roles in acceleration of COVID-19 vaccine development and we have summarized bioinformatics tools which are critical for vaccine development in Table 2. These tools are reviewed in details by others [19], [20].

**Table 2:** Bioinformatics tools for COVID-19 vaccine development used in the computational analyses, reduced from focused reviews [19], [20].

Number	Tool	Description
1	EpiMatrix	Maps T-cell epitopes across HLA class I and II
2	ClustiMer	Identifies promiscuous epitopes
3	Conservatrix	Identifies epitopes conserved across pathogen sequence variants
4	BlastiMer	Identifies epitopes with homology to autologous human proteins or to another organism of interest
5	EpiAssembler	Assembles overlapping epitopes to immunogenic consensus sequence
6	Optimatrix	Strategically alters peptides to optimize aggretope
7	Aggregatrix	Selects a set of peptides to maximize coverage of pathogen sequence variants
8	VaccineCAD	Minimizes “nonsense” immunogenicity at the junctions between epitopes in a string of beads construct
9	TepiTool	Provides some of the top MHC class I and class II binding prediction algorithms for number of species including humans, chimpanzees, bovines, gorillas, macaques, mice and pigs. The tool is designed as a user-friendly wizard with well-defined steps which helps the users to predict the best MHC binding peptides from their sequences of interest
10	PriSeT	Does computing of specific primers of SARS CoV2 for RT-PCR
11	CoVPipe	Helps in reproducible, reliable and fast analysis of NGS data
12	poreCov	It reduces the time-consuming bioinformatic bottlenecks in processing sequencing runs
13	VADR	It validates and annotates SARS CoV 2
14	V-Pipe	Reproduces NGS-based, end-to-end analysis of genomic diversity in intra-host virus populations
15	Haploflow	It detects and also does full-length reconstruction of multi-strain infections
16	VIRify	Identifies viruses in clinical samples
17	VBRC genome analysis tools	Visualizes the differences between coronavirus sequences at different levels of resolution
19	VIRULIGN	Fast, codon-correct multiple sequence alignment and annotation of virus genomes
20	Rfam COVID-19	It annotates structured RNAs in coronavirus sequences and predicts the secondary structures
21	UniProt COVID-19	Provides the latest information on proteins and its relevance to the disease for virus and host
22	Pfam	It detects protein and annotates for outbreak tracking and studying evolution
23	BLAST	It helps in detection of homologous regions of a given motif of vaccine candidate

Recently, Kumar et al. identified two peptide-based vaccine-like candidates in a rapid study of the multi-facet nucleocapsid (N) protein of SARS-CoV-2 using integrative bioinformatics approach [21]. Similarly, a new application of machine learning to predict antigen-specific immune signatures was developed [10]. AlphaFold (the deep learning system by Google DeepMind) has released predicted protein structures associated with COVID-19, valuable for COVID-19 vaccine formulation [22]. Another newly developed technology, Vaxign, a reverse vaccinology tool integrated with machine learning has also proposed COVID-19 vaccine candidates [23].

**Figure 2:**
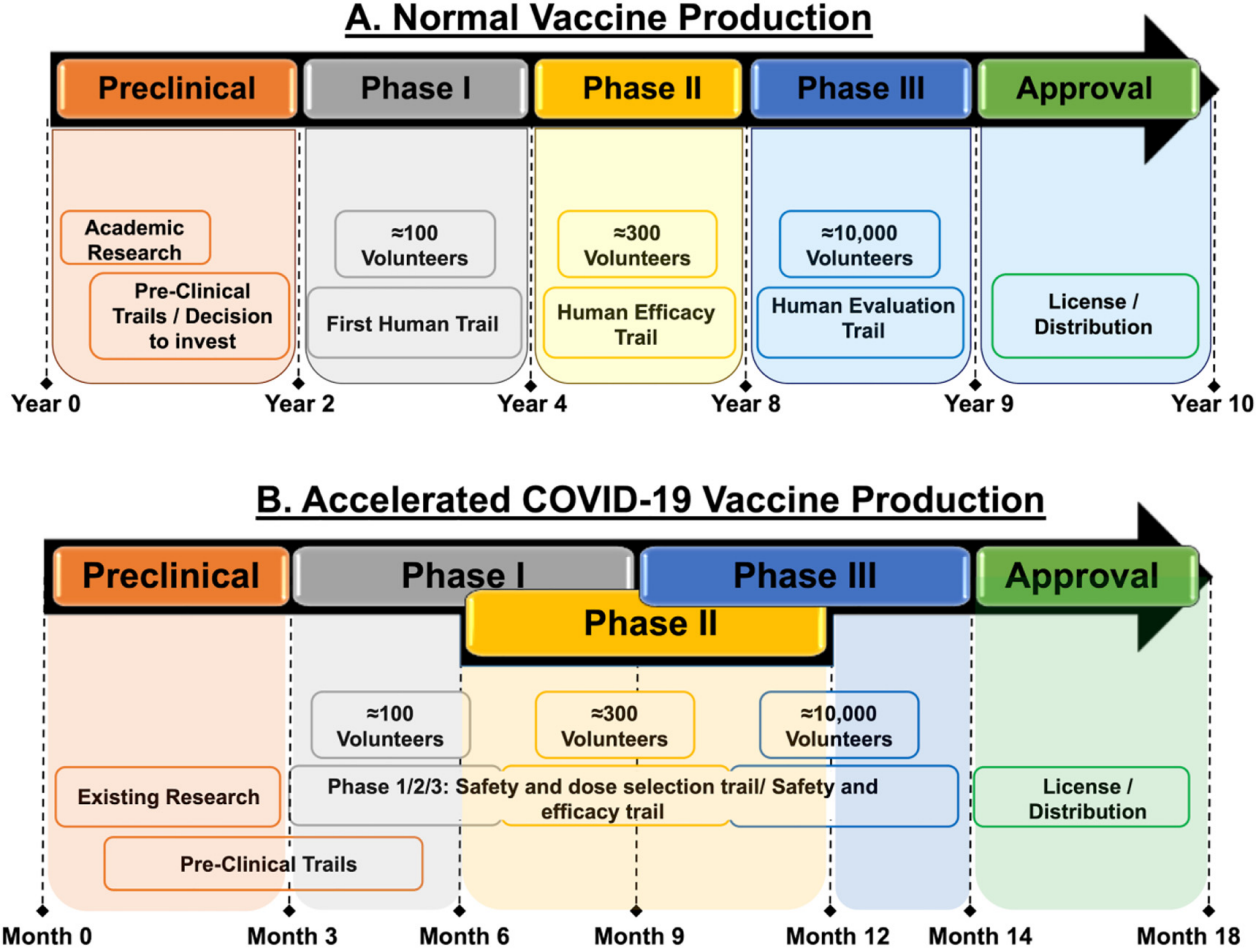
Phases of vaccine production in the normal (non-pandemic) versus accelerated pandemic COVID-19 threat. (A) Normally, it takes approx. 10 years for the approval of a single vaccine. (B) In the COVID-19 pandemic situation, the time period has been shortened to 18–20 months for the approval.

Parallel studies (Figure 2) of screening trials at the equivalent of the phase II and phase III trials have been initiated to reduce the time to develop a vaccine [24]. There has been an unprecedented level of global cooperation within the scientific community which has produced rapid results. The access to COVID-19 tools (ACT) Accelerator is one such groundbreaking global collaboration to accelerate development, production, and equitable access to COVID-19 tests, treatments, and vaccines.

Scientific communication has also stepped up in pace, for example, researchers have reported important findings on conference calls organized by WHO instead of the time-consuming process of writing and publishing academic papers [24].

Carrying out research and development (R&D) through the public sector but bringing in the private sector for manufacture and distribution approach has emerged as an aggressive strategy toward the vaccine. Initially, funding for the Jenner Institute’s COVID-19 research has come from various public and charitable sources and in April 2020, the Jenner Institute partnered with the British–Swedish company AstraZeneca for the development, manufacture and large-scale distribution of the vaccine on a not-for-profit basis, with only the costs of production and distribution being covered [24].

### 2.4 SARS-CoV-2 vaccine strategies

Currently, several potential SARS-CoV-2 vaccine candidates have been designed owing to the open-source data availability as summarized by WHO [6] and we have summarized as country-wise affords in Supplementary Table S1. These vaccine candidates have been categorized into various types such as live attenuated, inactivated, vectored, subunit/conjugate, nucleic acid-based, and recombinant vaccines as described in Figure 1 and also by other groups [18], [25], [26], [27].

### 2.5 Conventional COVID-19 vaccination strategies

#### 2.5.1 Live attenuated vaccines

Live attenuated vaccines are derived from the viral particles weakened or inactivated by physical, chemical, or biological modifications of the SARS-CoV-2 viruses. These enable the immune system to recognize the antigens to mount an immune response against SARS-CoV-2 [18], [25], [26], [27]. These live attenuated viruses can result in lifelong immunity with just one or two doses. These viruses are developed by introducing hundreds of silent mutations in the virus genome resulting in codons that are under-represented in human cells. However, these can be rapidly produced in cell cultures and formulated into vaccines within week’s time. These viruses upon administration, can infect the human cells but cannot reproduce faster giving the immune system sufficient time to mount an immune response and generate memory cells. Codagenix (USA) in collaboration with Serum Institute of India has developed a live attenuated COVID-19 vaccine by using codon deoptimization of the live virus [10], [28].

TNX-1800, from Tonix Pharmaceuticals is a live attenuated horse pox virus against COVID-19 [29]. The candidate vaccine has enrolled its first human participant in October under the PRECISION trial [30].

#### 2.5.2 Inactivated vaccines

Inactivated vaccines use antigenicity of the dead cells of the SARS-CoV-2 virus that have been killed with heat or chemical treatment [18], [26], [27]. The antigens on the dead viruses trigger the immune system and can be used in fighting against the live SARS-CoV-2 viruses. These vaccines can be whole inactivated viral particles or chemically modified specific components obtained from the SARS-CoV-2 viruses (called split virus vaccines). An adjuvant component can be used to enhance the immunogenicity of the inactivated viruses. However, the integrity/stability of the antigen or epitope needs to be confirmed prior to its development as a vaccine.

China’s CoronaVac vaccine from Sinovac company has been developed by inactivation of the SARS-CoV-2 by formaldehyde in the presence of alum as adjuvant [10]. In June, CoronaVac which was funded by Sinovac Research and Development Co., Ltd. plotted a pivotal vaccine trial in Brazil after a positive COVID-19 vaccine phase II trial [30]. The São Paulo state government in Brazil has requested from Brazil’s national health regulator, Anvisa, to register CoronaVac for inoculation and the national regulator received the first batch of data covering CoronaVac [30].

A joint venture by Wuhan Institute Products Co. and China’s National Pharmaceutical Group (Sinopharm) has developed an unnamed inactivated vaccine and has already dosed 2000 healthy volunteers with this unnamed COVID-19 vaccine. Another COVID-19 vaccine, BBIBP-CorV, by Beijing Institute of Biological Products, affiliated to China National Pharmaceutical Group (Sinopharm) has been successful in animal trials [31]. Phase I and phase II trials have been done for these two COVID-19 vaccines in China but are not qualified for phase III trials as the infection rates in Beijing are very low. Phase III trials are likely to be held in collaboration with Brazil if phase II trials showed successful results. On June 11th, 2020, the Philippine Department of Science and Technology and Beijing-based biotechnology companies are in talks on the phase III trials of these COVID-19 [30].

**Covaxin**: Bharat Biotech International Limited (BBIL), Hyderabad, in collaboration with the Indian Council of Medical Research (ICMR), New Delhi, and National Institute of Virology (NIV), Pune, has developed India’s first potential indigenous inactivated COVID-19 vaccine, Covaxin. BBIL started the first phase of testing on 375 healthy volunteers and these individuals aged >18 years and <55 years, with no co-morbidities and without a history of COVID-19 were recruited to participate in the randomized, double-blind, placebo-controlled clinical trials at various institutes and hospitals from India. The phase I and phase II human trials of this vaccine started on 15th July 2020 and testing of its efficacy and safety was performed in 12 institutes from India, selected by ICMR, New Delhi. The All-India Institute of Medical Sciences (AIIMS), Patna, conducted human trials in 10 healthy volunteers and has observed that the individuals did not have any adverse effects [32]. BBIL has got the approval to conduct phase III trials and recently has entered into an agreement with ViroVax, a Kansas-based company to use and license an adjuvant called Alhydroxiquim-II to enhance the vaccine by boosting the immune response and improving its efficacy [30]. To date, there are no reports of observable side-effects in the participants enrolled in the Covaxin vaccine development study.

#### 2.5.3 Replicating viral vectored vaccines

Viral vectored vaccines utilize the strategy of delivering one or more antigens encoded in a modified live attenuated or killed virus that expresses the antigens in the host system upon their delivery and thus lead to the immediate induction of the immune response [12]. These vectored vaccines can be either replicating viral vectors or replication-deficient viral vectors. Replicating viral vectored vaccines use the attenuated viral vectors that retain their replication ability and further enhance the induction of both cellular and humoral immune response. The most common examples for such vaccines are human immunodeficiency virus (HIV), vesicular stomatitis virus (VSV), *Sendai virus* and replication-competent adenoviral vector-based (Ad4 & Ad7) vaccines [33]. A replicating viral vector vaccine from The Institut Pasteur based on measles vector technology, CoroFlu influenza virus from University of Wisconsin and an influenza vector expressing RBD vaccine from University of Hong Kong are in pre-clinical trials [29]. For vaccine manufacturing, these viruses are either produced in cell lines that complement the missing replication machinery or the viral replication machinery itself is engineered such that these vectors cannot replicate in their full capacity. These are generally encoded in multiple plasmids to avoid encapsulation of these replication competent genes into the viral particles. Thus, these vaccines do not result in causation of the infections in the humans. At commercial scale, these vaccines are produced in suspension cultures. A single dose of these vaccines is reported to elicit strong immune responses in the host system [34].

#### 2.5.4 Conjugate/subunit or recombinant protein vaccines

Certain microorganisms consist of several microbial antigenic components that are coated with-in the subunits of protein or carbohydrates which help them to evade the human immune system. Subunit vaccines are composed of these protein or carbohydrate moieties conjugated with other antigens from the non-infectious recombinant proteins or synthetic peptides, such that the immune system will be activated to recognize the outer carbohydrate/protein subunits and produce antibodies against it. For instance, the target protein like S-protein can be directly purified from the virus or viral-infected cells as well [34]. Zydus Cadila, an Indian pharmaceutical company, has developed a vectored SARS-CoV-2 vaccine by using the live attenuated recombinant measles virus (rMV) [28], [35].

The firm has started phase I and phase II human clinical trials of its COVID-19 candidate ZyCoV-D which is a non-replicating and non-integrating plasmid DNA vaccine designed and developed by Zydus Universe and partially funded by the Department of Biotechnology (DBT), New Delhi. Zydus Cadila will enroll over 1,000 subjects across multiple clinical study sites in India [25], [36]. Currently, ZyCoV-D is in phase II stage.

#### 2.5.5 Stabilized subunit vaccines

The University of Queensland has been developing a stabilized subunit vaccine based on the molecular clamp technology that allows recombinant viral proteins to remain stable in their pre-fusion form. The University of Queensland has applied GSK’s adjuvant system for the development of an effective vaccine and entered a partnership with Coalition for epidemic preparedness innovations (CEPI [37]).

#### 2.5.6 Recombinant subunit vaccines

Clover Biopharmaceuticals is developing a recombinant subunit vaccine based on the trimeric S protein (S-Trimer) of the SARS-CoV-2. Clover Biopharmaceuticals and GSK announced a partnership to improve immune response by introducing GSK’s adjuvant system to S-Trimer [37].

At present, SARS-CoV-influenza recombinant subunit vaccine from Sanofi is in the pre-clinical stage and another gp-96 subunit vaccine program was announced in March 2020 by Heat Biologics [29]. It stepped into promising preclinical testing by August 2020 [6].

PittCoVacc is a novel microneedle array-based vaccine from University of Pittsburgh’s research that is in preclinical testing in mice [6]. Vaxart’s oral tablet formulation of a recombinant COVID vaccine is in the phase I stage [6].

#### 2.5.7 Trained immunity-based vaccines

The baseline tone of innate immunity can be increased by exposure to selected vaccines, such as bacille Calmette–Guérin (BCG) or microbial components, and induce antimicrobial resistance. This is called trained innate immunity. Such training is under trial against Covid-19 [38].

Researchers from Australia and UMC Utrecht & Radboud University, Netherlands have performed phase I trials with BCG vaccine in 4000 healthcare workers. Phase III trials are ongoing to evaluate the efficacy of this vaccine in COVID-19 children from various hospitals in Western Australia (NCT04327206). UMC Utrecht and Radboud University, Netherlands are investigating the efficacy of BCG vaccine in reducing absenteeism among healthcare workers involved in COVID-19 patient care (NCT04328441). The results of these studies are expected by the end of 2020 [31]. At present, WHO does not recommend BCG vaccination for the prevention of COVID-19 infections (WHO Newsroom, who.int/newsroom).

## 3 Innovative COVID-19 vaccination strategies

### 3.1 Non-replicating viral vectored vaccines

The non-replicating vectored vaccines utilize other weakened vectors for SARS-CoV-2 proteins such as the chimpanzee adenovirus and train the immune system to identify and combat the COVID-19 disease [27]. The spike (S) protein of SARS-CoV-2 is the common target for these viral vector-based COVID-19 vaccines. Johnson & Johnson’s company (New Brunswick, NJ, USA) and Altimmune Inc. (Gaithersburg, MD, USA) has developed an intranasal, recombinant adenoviral-based vaccine to stimulate the immune system and its human trial is planned in July. This vaccine candidate has been tested in 1045 healthy individuals between age 18–55 years in the US & Belgium and is currently in talks with the National Institutes of Allergy and Infectious Diseases (NIAID) [25]. Final-stage testing of Johnson & Johnson’s COVID-19 vaccine study has been paused when a study participant developed an unexplained illness subsequent to receiving the vaccine dosage [30].

### 3.2 ChAdOx1 nCoV-19 vaccine or AZD1222

ChAdOx1 nCoV19, an adenovirus vaccine vector, which is a weakened version of a common cold virus found in chimpanzees, is developed by the Clinical Biomanufacturing Facility at the University of Oxford Jenner Institute, UK. This vaccine uses a replication-deficient chimpanzee adenovirus, to deliver a SARS-CoV-2 spike protein to induce a protective strong immune response and inhibit the replication of the virus [31]. This vaccine contains the genetic sequence of the full length COVID-19 surface spike protein which causes the immune system to attack the SARS-CoV-2 virus with a human tPA leader sequence [39]. A study led by the Doremalen research group showed that a single vaccination with ChAdOx1 nCoV-19 effectively prevents damage to the lungs upon high dosage challenge with SARS-CoV-2. The same research group also proposed that this vaccine prevents the viral replication in the lower respiratory tract as the viral load is significantly reduced in BAL fluid and lung tissues of vaccinated animals [40]. At present, Oxford University has entered into a partnership with UK-based pharmaceutical company AstraZeneca for further development of the vaccine, its large-scale manufacture and potential distribution. To assess the vaccine efficacy, stability and immune response against the COVID-19 virus, pre-clinical and clinical trials [41].

#### 3.2.1 Pre-clinical vaccine trials

Testing of the ChAdOX1 nCOV-19 vaccine on animals was done in collaboration with Rocky Mountain Laboratories (NIAID/NIH) on rhesus macaque model to investigate the good safety and efficacy by inducing good immune response. Animal studies are also being done in Australia and the UK.

#### 3.2.2 Clinical vaccine trials

**Figure 3:**
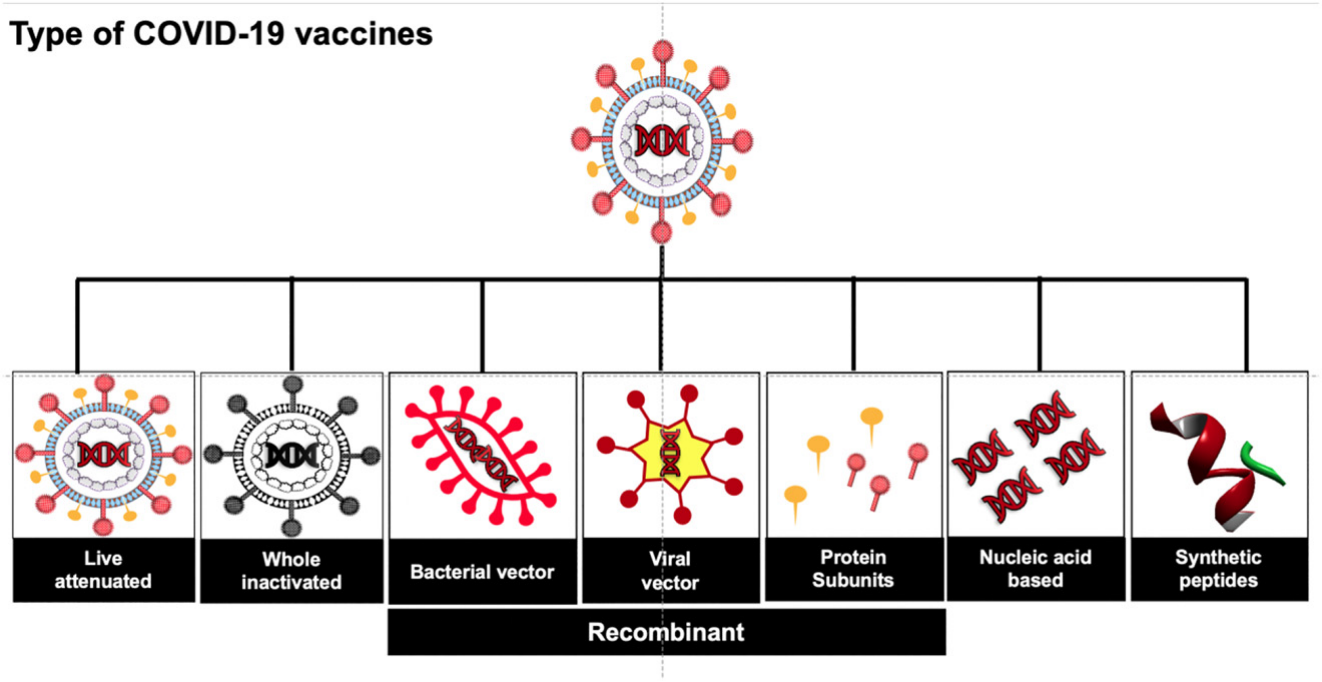
Types of COVID-19 vaccines.

Phase I trials began in April 2020, in healthy adult volunteers. More than 1000 immunizations have been completed and these individuals are being followed up as described in Figure 3B.

Phase II trials have enrolled up to 10,260 healthy adults and children and involved several partner institutions across the country. These phase II trials have expanded the age range of healthy volunteers for the study and included the volunteers aged: 56–69, over 70 and between 5 and 12 years to assess the variation in the immune response to the vaccine in older people or children.

Phase III trials have enrolled many people over the age of 18 years, to assess the safety aspects & efficacy of the vaccine, further, to prevent people from becoming infected & unwell with COVID-19. Randomized adult participants in both phase II and III groups will be receiving one or two doses of either the ChAdOx1 nCoV-19 or a licensed vaccine (MenACWY as an active control). Vaccinations have taken place in May or June 2020 and results are expected by mid-2021 [42] as explained in Figure 3. Currently, the Serum Institute of India (Pune, India) has collaborated with AstraZeneca for the phase III clinical trials (see Figure 2) of the AZD1222 vaccine in India and its distribution throughout the country as ‘Covishield’ [30]. However, according to a recent report [30], the final phase III clinical trial of the AZD1222 vaccine has been on hold temporarily in the U.S. as a female participant has developed severe neurological complications relating to the symptoms of transverse myelitis.

### 3.3 Ad5-nCoV vaccine from China

China’s CanSino Biologics along with the Beijing Institute of Biotechnology developed an adenovirus-based COVID-19 vaccine, AD5-nCoV to deliver genetic material encoding SARS-CoV-2 spike protein to the cells; the produced spike protein travels to lymph nodes and directs the immune system to create the antibodies against the spike protein [31]. The phase I/II trials were done in March 2020 to investigate the safety, reactogenicity, and immunogenicity in healthy individuals between 18 and 60 years of age through receiving the vaccine as an intramuscular injection (NCT04313127). In the May 2020, Wei Chen from the Academy of Military Medical Sciences, China, and CanSino Biologics entered into a co-development agreement with Vancouver, a British Columbia-based Precision NanoSystems for an mRNA lipid nanoparticle vaccine, Ad5-nCov against COVID-19 [41], [43]. The National Research Council of Canada in collaboration with CanSino Biologics Inc., to advance the bioprocessing and clinical development, has started the first Canadian clinical trials at the Canadian Centre for Vaccinology at Dalhousie University. Currently, this vaccine was approved, and its use is primarily limited to only military use for a period of one year by the Central Military Commission since June 2020. In mid-July, CanSino Biologics is in talks to launch a phase III trial of Ad5-nCoV, in collaboration with Russia, Brazil, Chile, and Saudi Arabia [30].

Gamaleya Research Institute is conducting phase II trials (NCT04587219) with Adeno based Gam-COVID-Vac in Moscow [44], while Janssen Pharma is in phase I with Ad26 vector based vaccine [45].

### 3.4 Nucleic acid-based (DNA/mRNA) vaccines

Nucleic acid-based (NA) vaccines consist of the specific genetic constructs of SARS-CoV-2 that can express the antigens like spike protein or other viral-specific components. These NA-based vaccines can be either DNA or RNA (mRNA) vaccines and can result in the activation of both humoral and cellular immune responses [12], [34].

### 3.5 DNA vaccines

Inovio Pharmaceuticals (Plymouth Meeting, PA, USA), in collaboration with Beijing Advaccine Biotechnology, China, has started pre-clinical trials of INO-4800, a DNA vaccine that is funded by Bill and Melinda Gates Foundation. Currently, phase I trials for the INO-4800 vaccine against COVID-19 are in progress, and its administration route is via intradermal delivery using electroporation. INO-4800 uses the spike gene of SARS-CoV-2 and induces activation of T cells by delivering DNA plasmids that express the SARS-CoV-2 spike [37]. Codagenix, in collaboration with the Serum Institute of India, has used a reverse strategy to create a live-attenuated vaccine CdX-005 by changing the optimized codons with non-optimized codons. The company is on course to initiate a phase I first-in-human clinical trial in the UK by the end of 2020 [30]. Similar vaccines are in pre-clinical testing by others like LineaRx and ZydusCadila. Karolinska-Cobra Biologicals and Genexine Consortium [29].

### 3.6 mRNA vaccines

#### 3.6.1 mRNA-1273 from Moderna Inc

A synthetic RNA-based vaccine, mRNA-1273 from Moderna Inc. is currently in phase III trials in partnership with the US National Institute of Allergy and Infectious Diseases (NIAID). A novel lipid nanoparticle (LNP)-encapsulated mRNA-based vaccine, mRNA-1273 encodes a full-length, prefusion stabilized spike protein from the SARS-CoV-2 virus. The phase I trials of this vaccine have been started by Emory University in Atlanta on 45 healthy individuals aged between 18 and 55 years on 16 March 2020 in collaboration with NIH [46]. 100 µg has been selected as the optimal dose level from phase I trial data to maximize the immune response and minimize adverse reactions [43].

The phase II and phase III studies of mRNA-1273 planning and execution are supported by the funding from BARDA within the US Department of Health and Human Services. For phase II trials, Moderna Inc. has enrolled 600 healthy individuals aged 18 and above (NCT04283461). The enrollment of healthy volunteers is going on at Emory Vaccine Center in Georgia, NIH, Maryland, and Kaiser Permanente Washington Health Research Institute, Washington, USA. Currently, enrollment of 30,000 participants is being done in collaboration with the NIH, USA, with the primary goal to prevent symptomatic COVID-19 & severe SARS-CoV2 infection. The production of required vaccine doses is done and phase III trials in 105 adults aged 18–99 years commences from July 2020 and is expected to be completed in 2021 in 3000 subjects at 100 µg dose level. This vaccine is designed *in silico* rather than a cell culture-based system to enable the rapid development and evaluation of vaccine efficacy [37], [43]. The scale-up of mRNA-1273 manufacturing is being planned both at Moderna Inc. and its strategic collaborator, Lonza Ltd [32]. The early-stage human trials in 45 patients, has shown that Moderna’s mRNA-1273 produced neutralizing antibodies in all the patients. For these early phase III human trials 45 volunteers recruited for the study were categorized as three dose groups and have received two doses of either 25 or 100 or 250 µg, with 15 people in each group. The levels of neutralizing antibodies in patients in the high dose group i.e., 100 and 250 µg were two-fold and four-fold higher than in recovered COVID-19 patients respectively [32].

#### 3.6.2 BNT162 vaccine from China

BioNTech SE (Mainz, Germany) in collaboration with Pfizer Inc. (New York, NY, USA) and FoSun Pharma (Shanghai, China) is developing and evaluating four experimental COVID-19 vaccines: BNT162a1, BNT162b1, BNT162b2, and BNT162c2 [41]. Both BNT162a1 and BNT162b2 are nucleoside-modified mRNAs (modRNA), the other two candidates are a uridine containing mRNA (uRNA) and a self-amplifying mRNA (sRNA). All mRNA candidates are developed by encapsulating the nucleic acids and formulated in special 80 nm ionizable, glycol-lipid nanoparticles (LNPs). BNT162b1 encodes an optimized SARS-CoV-2 receptor-binding domain (RBD) antigen, while BNT162b2 encodes an optimized SARS-CoV-2 full-length spike protein antigen [32]. Both BNT162b1 and BNT162b2 received FDA’s Fast Track designation based on preliminary data from currently ongoing phase I/II clinical trials (NCT04368728) in the U.S. and Germany as well as animal immunogenicity studies. The volunteers aged between 18 and 55 years received two doses each, in the range of 10–30 µg and the optimal dose will be determined depending on the data from these studies [32]. This study showed that the neutralizing titers were found to be 1.8–2.8-fold in comparison to that of a panel of COVID-19 recovered patients’ sera. Enrolment of up to 30,000 healthy participants is being planned for a global phase IIb/III clinical trials from July 2020 [32].

CureVac’s CVnCoV is another mRNA vaccine developed in Germany, which has shown promising results in phase I trials [47] and LNP-nCoVsaRNA by Imperial College London moves on to phase II clinical trials in The United Kingdom [48]. Two LNP-encapsulated mRNA cocktail vaccines are being tested in by Fudan University and one mRNA onco vaccine is being tried by BIOCAD in the preclinical stage [29].

### 3.7 Artificial antigen-presenting cells (aAPC) vaccine

A research group from Shenzhen Geno-Immune Medical Institute, China developed an acellular aAPCs vaccine which is a promising immunotherapeutic agent that can stimulate and amplify antigen-specific CD4+ T cells. The primary aim of this vaccine is to immune reactivate the T-cells to treat and prevent COVID-19. Phase I trials have been in progress with recruitment of healthy volunteers from the age of six months to 80 years in China (NCT04299724; NCT04276896). This study is expected to be completed in 2023 and 2024 [31].

### 3.8 Lentiviral (LV-SMENP-DC) vaccine & antigen-specific cytotoxic T lymphocytes (CTLs)

LV-SMENP, an innovative approach has been developed using COVID-19 minigenes engineered into a vaccine developed by the Shenzhen Geno-Immune Medical Institute, China. LV-SMENP is developed from multiple genes through the lentiviral vector system (NHP/TYF) to express COVID-19 antigens, causing dendritic cell (DC) modifications and T-cell activation. Currently LV-SMENP is undergoing phase I/II multicenter trial in healthy volunteers and COVID-19 infected patients aged between >6 months and 80 years individuals (NCT04276896). The results are expected by 2024 [31]. Shenzhen Geno-Immune Medical Institute developed two vaccines (LV-SMNEP-DC and aAPC) in clinical trials based on dendritic cells and antigen-presenting cells modified by lentiviral vectors expressing portions of the SARS-CoV-2 genome [36].

## 4 Other COVID-19 vaccines in clinical trials

### 4.1 Thailand vaccine

The National Primate Research Center in Thailand had developed a COVID-19 vaccine and the first dose had been given to macaque monkeys on 23rd May 2020 which showed satisfactory immune response. Same group of monkeys were administered a second dose of the vaccine in June 2020. Human trials will commence from October or November depending on the safety and efficacy of the vaccine [32].

### 4.2 Vaccine from Nigeria

Adeleke University in Osun, Nigeria, and Precious Cornerstone University collaborated to develop a vaccine locally for Africans. It takes another 18 months for the vaccine to be available to the public [32].

Encapsulated or covalent functionalized nanoparticles are conjugated with antigenic epitopes, mimic viruses called virus-like particles, and provoke antigen-specific lymphocyte proliferation and cytokine production. Novavax, Inc. is involved in producing a nanoparticle-based vaccine NVX-CoV2373 using antigens-derived from the coronavirus S protein. The S protein with a Matrix-M adjuvant is now in phase III trials.

HaloVax is testing a self-assembling technology (VaxCelerate) based vaccine in preclinical experiments. VaxCelerate combines a fixed immune adjuvant and variable immune targeting to elicit an immune response against SARS-CoV-2 [6].

Panacea Biotech has established a partnership with US-based Refana Inc. and is in preparation for 500 million doses of the inactivated virus-based vaccine. But Panacea Biotech has terminated the MOU for the development, manufacture, and supply of a vaccine for COVID-19 in collaboration with Refana [49].

Genexine Inc. is developing a vaccine using proprietary long-acting platform Hyleukin-7 platform (hyFc) technology. Interleukin-7 (IL-7) is fused to hyFc and is called GX-I7. It is designed to reinvigorate persistent T cell immunity by increasing the lymphocyte count of patients [37]. NeoImmuneTech (NIT) received FDA approval to conduct phase I clinical trials assessing GX-I7 as a treatment for adults with mild COVID-19 [50].

## 5 Passive immunisation against COVID-19

In passive immunization, preformed or manufactured antibodies to the SARS CoV2 virus are administered into the patients. These antibodies may be recovered from the acellular portion of the blood of patients who have recovered from COVID-19 (convalescent plasma) or by manufacture of the antibodies.

### 5.1 Convalescent plasma

Blood of patients who have recovered from COVID-19 has been processed to collect the plasma (which contains the antibodies) and was administered to COVID-19 patients. It has been shown to be beneficial in these cases. But there have been no randomized controlled trials to make the evidence strong [51].

### 5.2 Monoclonal antibodies

Monoclonal antibodies are antibodies against SARS CoV-2 that are manufactured by various techniques [52]. These have yet to show any therapeutic potential. Recently Eli Lilly’s antibody treatment started by a US-based health care center, LY-CoV555, AbCellera Biologics was found ineffective [53].

## 6 Challenges ahead

There are some challenges ahead as the vaccines roll out to the general public. On the manufacturing front, effective removal of impurities, maintaining high recovery yield, and reducing the price per dose are the important factors. These can be addressed by optimization of downstream process like centrifugation, clarification, ion exchange chromatography, size exclusion chromatography, DNA digestion, and tangential flow filtration are the common methods for downstream processes [54].

The mode of administration of the majority of these vaccines can be either intramuscular, subcutaneous or intradermal routes. Several research groups are also working on the development of the intranasal, inhalation-based and oral thermostable solid vaccines for COVID-19 [55]. Even though several studies have highlighted that the route of administration plays a significant role in enhancing the vaccine’s efficiency, stability, effectiveness and the immediate production of immune response in the patients, the best route of COVID-19 vaccine is yet to be investigated [10].

Vaccine targets may cause a wide range of side effects. These include the induction of cytokine storms (i.e., IL6), lung immunopathology, hepatitis, hemotoxicity, cytotoxicity, cross-reactive antibodies, and allergenicity. Minimizing the size of vaccine candidates from protein to epitope/peptide will overcome the side effects. Efforts are also being made to improve heat stable vaccines that can address the challenges of transportations and cold storage to reach long distance populations. Novel technologies such as implants, microneedle patches [56] and lyophilised preparations [10] may be solutions against the problems of temperature stability and transport.

Also, the clarity of its effectiveness in the population in a clinical setting and availability to billions of people at accessible cost need to be addressed. A rationalized vaccine administration paradigm may be needed in the limited available phase of a successful vaccine where high-risk populations such as health-care workers, seniors and people with co-morbidities would be given priority [57].

At present, these are the major challenges faced by the scientific communities, pharmaceutical companies and global leaders working the development of COVID-19 vaccines. One answer is the COVAX (The COVID-19 Vaccines Global Access Facility) under the ACT umbrella which is a global collaboration of 78 countries for speeding up the development, manufacture and equitable distribution of new vaccines. COVAX supports research and development of new vaccines by investing in them and negotiating prices with pharmaceutical companies. All participating countries, regardless of income levels, will have equal access to these vaccines once they are developed. The goal of COVAX is to have two billion doses to distribute by the end of 2021, which should be enough to help countries vaccinate 20% of their populations and end the acute phase of the pandemic [58].

## Supplementary Material

Supplementary Table S1. List of country-wise affords of COVID-19 vaccine developments.
